# The burden of firearm violence in the United States: stricter laws result in safer states

**DOI:** 10.5249/jivr.v10i1.951

**Published:** 2018-01

**Authors:** Faisal Jehan, Viraj Pandit, Terence O’Keeffe, Asad Azim, Arpana Jain, Saad A. Tai, Andrew Tang, Muhammad Khan, Narong Kulvatunyou, Lynn Gries, Bellal Joseph

**Affiliations:** ^*a*^Department of Surgery, Division of Trauma, Critical Care and Emergency Surgery, University of Arizona, Tucson, AZ.

**Keywords:** Firearm injuries, Legislation

## Abstract

**Background:**

Increasing firearm violence has resulted in a strong drive for stricter firearm legislations. Aim of this study was to determine the relationship between firearm legislations and firearm-related injuries across states in the United States.

**Methods:**

We performed a retrospective analysis of all patients with trauma related hospitalization using the 2011 Nationwide Inpatient Sample database. Patients with firearm-related injury were identified using E-codes. States were dichotomized into strict firearm laws [SFL] or non-strict firearm laws [Non-SFL] states based on Brady Center score. Outcome measures were the rate of firearm injury and firearm mortality. Linear Regression and correlation analysis were used to assess outcomes among states.

**Results:**

1,277,250 patients with trauma related hospitalization across 44 states were included of which, 2,583 patients had firearm-related injuries. Ten states were categorized as SFL and 34 states as Non-SFL. Mean rate of firearm related injury per 1000 trauma patients was lower in SFL states (1.3±0.5 vs. 2.1±1.4; p=0.006) and negatively correlated with Brady score (R2 linear=-0.07; p=0.04). SFL states had a 28% lower incidence of firearm related injuries compared to Non-SFL states (Beta coefficient, -0.28; 95% CI, -1.7- -0.06; p=0.04). Firearm related mortalities resulted in overall 9,722 potential life years lost and more so in the non-SFL states (p=0.001).

**Conclusions:**

States without SFL have higher firearm related injury rates, higher firearm related mortality rate, and significant potential years of life lost compared to SFL states. Further analysis of differences in the legislation between SFL and non-SFL states may help reduce firearm related injury rate.

## Introduction

The United States ranks number 1 in the list of countries with most privately owned guns with 101 guns for every 100 individuals.^1^ This has resulted in the loss of 32 lives and the treatment of 140 people every single day for gun related violence.^2^ These numbers state that there are more lives lost in seven weeks at the hands of firearm related violence than the total number of lives lost in seven years of Iraq War.^3^ Firearm related deaths disproportionately involve the younger population resulting in premature deaths. Along with the loss of life, gun violence related injuries exert a major burden on the US health system costing up to $2.3 billion annually.^4^ The overall economic burden associated with gun violence actually exceeds more than $100 billion every year.^5^

The Second Amendment to the constitution of the United States was passed in 1791 as a part of the Bill of Rights, which protects the rights of individuals to keep and bear firearms. Ever since then, possession of firearms has been a matter of debate due to concerns for public safety. Lawmakers that tried to limit the access of firearms have met with criticism for violating the rights of citizens protected by the Second Amendment. Despite the hue and cry for needing stricter firearm laws, there has been limited legislative progress on this forefront since the Gun Control Act of 1968 following the Kennedy assassination. The shooting of Arizona Congresswoman Gabriel Gifford in 2011 and the mass shooting of Sandy Hook Elementary School in 2012 once again surfaced the debate on need for stricter firearm laws.

Limited numbers of previous studies have assessed the association between the incidence of firearm related mortality and strictness of firearm laws across the states.^6-8^ Every two years the Brady Campaign to Prevent Gun Violence issues a 100-point scorecard (Brady scorecard) that assigns a numerical value to each state based on the strength of its firearm laws. Brady scorecard was based on policy regulations such as background checks on gun sales; reporting lost or stolen firearms; and prohibiting dangerous people from purchasing weapons.^9^ The higher the Brady Score for each state, the stricter the firearm laws in that state.

We for the first time used the Brady scorecard to assess the level of strictness in firearm laws across different states. The aim of this study was to determine the association between the rigorousness of firearm laws across different states and the rate of firearm related injuries, and mortality. We hypothesized that strict firearm legislations lower the incidence of firearm related hospital admissions and mortality.

## Methods 

**Data source:**

Hospital admission data was obtained from the National Inpatient Sample (NIS) database for the year 2011. NIS is the largest public database maintained by the Healthcare Cost and Utilization Project (HCUP) containing information from more than 4000 hospitals and 7 million in-hospital admissions across 44 states in the United States for the year 2011. The database is a 20% stratified sample of all hospital admissions in the U.S. The data are weighted back to help make population estimates of the various parameters. The NIS database is the largest all-payer inpatient care database publically available in the United States. It covers 95% of the US population and includes comprehensive abstracted discharged data. NIS contains information like patient demographics, admission profile, state codes, admission months, discharge diagnosis, procedure codes, hospital charges and discharge time. For the accurate analysis of trends, we merged the Healthcare Cost and Utilization Project (HCUP) trend weight file onto the original NIS files by year and the HOSPID.

**Patient population:**

Trauma patients for the study were identified from the NIS database using the Ninth Revision of the International Classification of Diseases E-Codes (800-959). Patients with missing information on mechanism of injury and location of hospital were excluded.

**Data Points Collected:**

The following data points were collected from the database: patient demographics (age, gender and race), date of admission, mechanism of injury, hospital length of stay, hospital charges, and in-hospital mortality. Patients with firearm related injuries were identified using the following E-Codes: suicides or suicide attempts (E955.0, E955.2, E955.3 and E955.4), assaults (E965.0-E965.3) and unintentional (E922.0-E922.4).

**State firearm legislations and scoring:**

Brady Center to Prevent Gun Violence and Brady Campaign to Prevent Gun Violence brought forward a scoring method to evaluate and rank the strength of gun laws and policies for all 50 states across the United States. Sixteen gun violence prevention policies were evaluated. These can be broadly classified into 5 categories: (i) Firearm purchase, (ii) Background checks, (iii) Assault weapons, (iv) Child safety and (v) Guns in public places. Table 1 describes the Brady scoring system. Each individual policy is assigned a score based on its effectiveness to reduce gun violence. Each state is given a cumulative Brady score out of 100, which is calculated by adding the scores for all policies present in the state. The higher the total aggregate score for each state, the stricter the firearm laws in that state. The state scorecard is updated every year and takes into account the changes in gun and ammunition laws in each state every year. The Brady state scorecard for 2011 was used for our study. ^9^

**Table 1:** Brady scoring system.

**Curb firearm trafficking (35 points)**
Gun dealer regulations (12 points)
Limit bulk purchases (5 points)
Record retention (5 points)
Crime gun identification (10 points)
Report Lost/Stolen guns (3 points)
**Strengthen Brady background checks (40 points)**
Background checks on all gun sales (17 points)
Permit to purchase (21 points)
Ammunition record (2 points)
**Ban assault weapons (10 points)**
Assault weapons ban (5 points)
Large capacity magazine ban (5 points)
**Child safety (7 points)**
Child safety locks (5 points)
Child access prevention (2 points)
**Guns in public places and local control (8 points)**
No guns in work place (2 points)
No guns on college campuses (2 points)
Not a CCW shall issue state (2 points)
No state preemption (2 points)

We calculated the median score for all the states. The median states’ scores were bimodaly distributed. The intersection between two normal distributions (score of 26) was used as a cutoff to categorize states as Strict Firearm Laws (SFL) and non-Strict Firearm Laws (non-SFL). All states with a Brady score of 26 or above were labeled as SFL while states with Brady score less than 26 were labeled as non-SFL. Thirty-four states were labeled as Non-SFL while 10 were labeled as SFL.

Primary outcome measure was incidence of firearm related injuries. Secondary outcome measures were firearm related mortality, hospital length of stay, and Potential years of Life Lost (PYLL). We calculated PYLL by subtracting the age of death for each patient from the reference age of 75 years. To calculate the total PYLL for SFL and non-SFL states, the individual PYLLs for all mortalities in each group were summed and were divided by the trauma population and are presented in the form of mean with the standard deviation.

**Statistical Analysis:**

Data are reported as mean ± standard deviation (SD) for continuous variables, as frequency and proportions for categorical variables, and as median [interquartile range] for ordinal variables. Incidence of firearm related injury and mortality are reported as rates of firearm related injury or mortality/1000 trauma patients. We performed chi-square test (categorical variables) and independent t -test (continuous variables) to compare demographics and outcomes between SFL and non-SFL. We performed Pearson correlation analysis to assess the correlation between Brady score and rate of firearm related injuries. A linear regression analysis was also performed to evaluate the impact of firearm laws on firearm related injuries. Beta coefficient and 95% confidence intervals were calculated for each variable in the regression model. To assess the goodness of fit of our linear regression model, the assumptions were placed on the residuals and estimated residuals were used including normal distribution, outliers and constant variance and the model was deemed fit. For our study, we considered p value of less than 0.05 as statistically significant. All statistical analyses were performed using Statistical Package for Social Sciences (SPSS, Version 21; SPSS, Inc., Armonk, NY).

## Results

**Figure 1:**
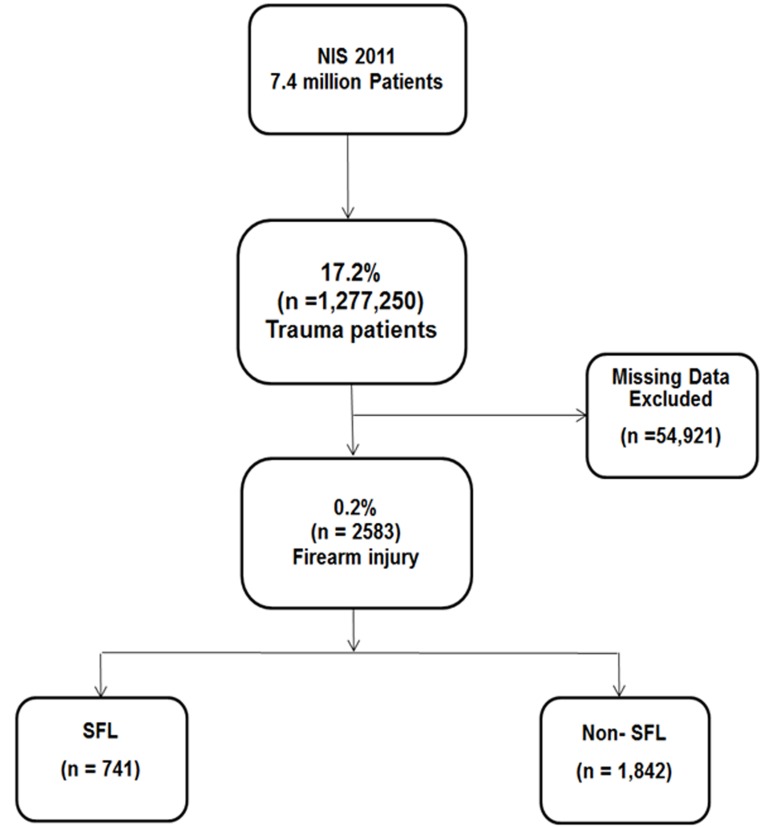
Study population.

A total of 7.4 million patients in the database were analyzed of which 17.2% (1,277,250) were trauma patients. The proportion of trauma patient in states with SFL and in those states with non-SFL was 15.9% and 18.3% respectively. Out of the total of 1,277,250 trauma patients, 4.3% had missing data on mechanism of injury or the hospital location and were excluded. 2,583 (0.2%) patients had firearm related injuries and were included (Figure 1). Of the 2583 patients, 810 are SFL states and 1,773 are non-SFL states. States with SFLs had lower mean rates of firearm related injuries per 1000 trauma patients as compared to states with non-SFLs (1.3±0.5 vs. 2.1±1.4; p=0.006). The mean age of our study population was 33.2±17.7 years, 87.8% (n=2,268) were male and 40.3% (n=1,041) were white. The demographics of our study population are shown in Table 2. Patients in the SFL states were younger in age (p<0.001), more likely to be male (p<0.001), and less likely belonged to white race (p<0.001) compared to patients from non-SFL states.

**Table 2:** Demographics.

Variables	SFL (n=810)	Non-SFL (n=1,773)	p value
Age, mean ± SD, years	30.3±17.6	34.6±17.5	>0.001
Male, % (n)	91.0% (737)	86.8%(1,540)	>0.001
Race			
White, % (n)	24.6% (199)	47.5% (842)	>0.001
Weekend admission, % (n)	36.8% (298)	34.0% (603)	0.18
Mechanism of injury			
Suicide, % (n)	13.5% (109)	27.7% (491)	>0.001
Assault, % (n)	68.8% (557)	40.2% (712)	>0.001
Unintentional, % (n)	17.8% (144)	32.3% (572)	>0.001

Bold cells represent statistically significant p-values

Overall 284/2583 (11.0%) died during the hospital stay from firearm related injuries. The in-hospital mortality rate from firearm related injuries was significantly lower in SFL states (67/810, 8.3%) as compared to non-SFL states (217/1773, 12.2%; p=0.002). The mean Hospital LOS was 6.5±10.8 days, and it did not differ between SFL states and non-SFL states (7.0±14.9 vs. 6.3±8.3; p=0.22). 

Overall Potential years of Life Lost (PYLL) from firearm related deaths were 9,722 years. The mean number of PYLL in SFL states were significantly lower (2.7±11.0 years) as compared to those in non-SFL states (4.4±14.6 years; p=0.001). Table 3 demonstrates outcomes of the study population.

**Table 3:** Outcomes.

Variables	SFL (n=810)	Non-SFL (n=1,773)	p value
Rate of firearm injury/1000 trauma admissions, mean ± SD,	1.3±0.5	2.1±1.4	0.01
Firearm mortality, % (n)	8.3% (67)	12.2% (217)	0.01
Hospital Length of stay, median [IQR]	3 [1-7]	3 [2-7]	0.22
Potential Years of Life Lost, mean ± SD,	2.7±11.0	4.4±14.6	0.001

Bold cells represent statistically significant p-values

**Figure 2:**
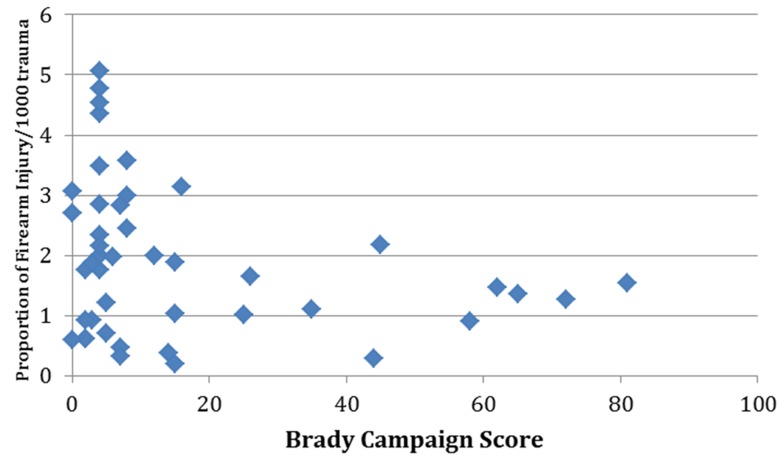
Correlation analysis between Brady score and rate of firearm related injuries.

On Pearson’s correlation analysis, the rate of firearm related injuries was found to be negatively correlated with Brady score (R2 linear=-0.07; p=0.04) and had a correlation coefficient of -0.265. Figure 2 shows the correlation analysis between Brady score and rate of firearm related injuries. On linear regression analysis, it was found that being in a SFL decreased the mean rate of firearm related injury by 28% (β coefficient, -0.28; 95% confidence interval, -1.7- -0.06; p=0.04). Table 4 demonstrates the results of linear regression analysis.

**Table 4:** Linear Regression Analysis for firearm-related injuries.

Variables	Beta [95% CI]	p value
Strict Firearm Law States	-0.28[-1.7- -0.06]	0.04
Age	0.04[-0.05-0.07]	0.78
Male	-0.08[-1.01-6.69]	0.14
White Race	-0.17[-0.02-0.01]	0.27
Weekend admission	0.05[-0.02-0.03]	0.73

Each variable represents a univariate regression analysis. Bold cells represent statistically significant p-values

## Discussion

The United States has one of the highest firearm related violence rates in the world overall; however great variability in individual rates exists across the different states. Numerous factors including differences in socio-economic factors, cultural differences and local firearm laws may account for these differences in firearm related injuries. Our study used the Brady scorecard; which provides an objective assessment of the rigorousness of state firearm laws and its impact on the rate of firearm related injuries and mortality. We found that states with SFL have a significantly lower number of firearm related hospital admissions and mortality when compared to non-SFL states across the United States. 

Following the year 2000, the number of firearm related injuries began to rise. Cook et al. studied all the gunshot wounds over the decade (2004-2013) and showed that the high rate of gunshot assaults on young black men and high suicidal firearms injuries in older white men persisted over the decade of this study.^10^ Amidst the increasing number of mass shooting incidents in the country, the rising media coverage and ongoing political debate, the number of publications related to firearms also began to increase with many of them looking at the impact of legislation to firearm related injuries. Ludwig et al. found that Brady Act resulted in reduction of suicide rates for 55 years and above but no change in overall suicide or homicide rates.^11^ Similarly, an age limit of 21 years for the purchase of handgun did not reduce the firearm related homicide or suicide rates.^11^ On the other hand some studies estimated the aggregate relationship of all state firearm laws with firearm related deaths. Fleegler et al. found that a higher number of state legislations were associated with a lower number of firearm related homicide and suicide rates.^6^ However; this study equally weighed all firearm legislations and did not take into account the impact factor of individual legislations on firearm related violence control. Similarly, Sumner et al. also demonstrated that performing local-level background checks before purchase of firearms was associated with a 27% lower firearm suicide rate and a 22% lower homicide rate.^12^ Most studies show that restrictions on firearms in states reduce the suicide rates.^12-14^ Besides legislature, safe gun practices that include keeping guns unloaded and ammunition locked at different locations have also shown to reduce firearm related injuries.^15,16^

We assessed the cumulative impact of firearm legislature strength on rates of firearm related hospital admissions and mortality across different states using the Brady scorecard. The strength of our study is that Brady scoring system takes into consideration several different aspects of firearm related policies like carrying weapons in the public, state bans on assault weapons and laws aimed at reducing gun trafficking. Brady scoring system awards different points to each law based on its individual weighted impact on violence protection. For example, any state with laws that required permit to purchase, safety training and finger printing would be awarded as many as 21 points while the mandatory reporting of a lost firearm would be awarded a mere 5 points. Our correlation analysis showed a negative correlation between the Brady score and rates of firearm related hospital admissions. Findings of our study do not prove direct causality for this association however; we believe that it does provide a broad sense of the impact of firearm laws. 

We also looked at the impact of Brady score on Potential Years of Life Lost (PYLL) from firearm related mortalities. PYLL provides an accurate measure of premature deaths from a cause predominant in the young, and helps to quantify the social and economic impact associated with it. Our population mainly comprised of young patients with a mean age of less than 40 years with significantly higher PYLL in non-SFL states as compared to SFL states. Our study for the first time provides evidence that SFL are not only associated with lower fatality but are also associated with significantly lower premature deaths that salvage many years of potential life. This finding unveils a significant aspect of stricter firearm legislation that has never been reported before.

Apart from these findings, we discovered some interesting demographic differences between the two groups. We found that states with stricter firearm laws had significantly lower number of female patients with firearm related injuries. In both SFL and non-SFL states, the most common mechanism of firearm related injuries was assault. However, the proportion of patients with suicidal and unintentional firearm injuries was significantly lower in the SFL-states compared to the SFL-states. These findings imply that stricter firearm legislations are associated with lower suicidal and unintentional firearm related injuries.

Our study has several limitations. First of all, NIS is a 20% sample of all inpatient discharges, weighted to represent national estimates. Abstracting the data on a state level might introduce a selection bias, as data is not weighted on a state level. However, similar methodology has previously been used and published in literature.^17,18,19^Our numbers relied on NIS database, which includes all patients admitted to the hospital with a diagnosis of firearm related injury but does not include patients that died before reaching the hospital. The differences in the implementation of firearm related laws across states could also not be taken into account.

Despite these limitations, our study sheds light on some important aspects of legislative strength and firearm related violence. We believe the findings of our study add to the growing literature on this issue and may provide an impetus for policy makers to find a solution for this growing menace, although it may be much easier said than done. States without strict firearm legislation have higher firearm related injury rates, higher firearm related mortality rate, and significant PYLL compared to SFL states. Further analysis of differences in the legislation between SFL and non-SFL states may help reduce firearm related injury rate.
